# Efficacy of Thermotherapy to Treat Cutaneous Leishmaniasis: A Meta-Analysis of Controlled Clinical Trials

**DOI:** 10.1371/journal.pone.0122569

**Published:** 2015-05-26

**Authors:** Jaiberth Antonio Cardona-Arias, Iván Darío Vélez, Liliana López-Carvajal

**Affiliations:** 1 Microbiologist and Bioanalyst, School of Medicine, Cooperative University of Colombia, Medellín, Antioquia, Colombia; 2 PECET, University of Antioquia, Medellin, Colombia; 3 Epidemiology, PECET, University of Antioquia, Medellin, Colombia; 4 School of Microbiology, University of Antioquia UofA, 70 Street # 52–21, Medellin, Colombia; The Ohio State University, UNITED STATES

## Abstract

**Introduction:**

The efficacy of thermotherapy for the treatment of cutaneous leishmaniasis presents diverse results with low statistical power.

**Objective:**

To evaluate the efficacy of thermotherapy to treat cutaneous leishmaniasis.

**Methods:**

A meta-analysis of controlled clinical trials in 12 databases based on the implementation of a research protocol with inclusion and exclusion criteria and an assessment of methodological quality. The reproducibility and completeness were guaranteed in the information search and extraction. Heterogeneity, sensitivity and publication bias were assessed by graphical methods (Galbraith, L'Abblé, funnel plot, Egger plot, and influence plot) and analytical methods (DerSimonian-Laird, Begg and Egger). Random-effects forest plots were constructed, and a cumulative meta-analysis was performed.

**Results:**

Eight studies were included with 622 patients who underwent thermotherapy, with an efficacy of 73.2% (95% confidence interval (CI) = 69.6-76.7%), and with 667 patients who underwent systemic treatment, with an efficacy of 70.6% (95% CI=67.1-74.1%). Heterogeneity between studies, good sensitivity for the combined measure, and no publication bias were observed. The relative risk for comparison of the efficacy of treatment was 1.02 (95%CI=0.91, 1.15), showing that the effectiveness of thermotherapy is equal to that of pentavalent antimonial drugs.

**Conclusion:**

Due to its efficacy, greater safety and lower cost, thermotherapy should be the first treatment option for cutaneous leishmaniasis in areas where the prevalence of the mucocutaneous form is low and in patients with contraindications to systemic treatment, such as kidney, liver and heart diseases, as well as in pregnant women, infants, and patients with human immunodeficiency virus infection/acquired immune deficiency syndrome.

## Introduction

The leishmaniases are zoonoses that affect the skin, mucous membranes or viscera, are caused by flagellate protozoa of the *Trypanosomatidae* family, genus *Leishmania*, with more than 20 species worldwide and are transmitted by phlebotomine insects [1].

This disease is endemic in tropical and subtropical regions of 98 countries [2] in Europe, Africa, Asia, and the Americas; in the latter continent, it has been reported from northern Argentina to southern Texas, except in Chile and Uruguay [1]. Leishmaniasis represents a serious and growing health problem worldwide due to its great extent [1], with an estimated 350 million people at risk worldwide, a prevalence of 12 million cases and an annual incidence of 2 million new cases, of which 1.5 million are cutaneous leishmaniasis [2,3]. Regarding mortality, data are limited, and estimates based on hospital records indicate a mortality of 10%, with approximately 20,000 to 40,000 deaths per year [4].

More than 90% of cases of visceral leishmaniasis were reported in six countries: India, Bangladesh, South Sudan, Sudan, Ethiopia and Brazil. Cutaneous leishmaniasis has a greater worldwide distribution, with about one-third of cases occurring in three regions: the Americas, the Mediterranean Basin, and western Asia from the Middle East to Central Asia. The 10 countries with the highest reported cases are Afghanistan, Algeria, Colombia, Brazil, Iran, Syria, Ethiopia, Northern Sudan, Costa Rica, and Peru, which correspond to 70–75% of the estimated incidence of cutaneous leishmaniasis in the world [4].

Some reports have indicated that these figures are underestimated due to the following factors: the distribution is not continuous in endemic areas, underdiagnoses are high, most of the official data come from passive case searches, the number of asymptomatic infected cases is high, and notification is mandatory in only 40 endemic countries [1].

From a clinical perspective, cutaneous leishmaniasis presents as papules that progress to nodules and ulcers whose clinical impact depends on the immune response, parasite species and time of infection [1]. In the ulcerative form, the disease generates distress, disability, loss of working time and wages, scarring and stigmas due to damage of the exposed areas of the body [5].

Treatment includes physical methods such as curettage, surgical excision, thermotherapy, cryotherapy and laser therapy; topical treatments with paromomycin and intralesional injections of pentavalent antimonials; and systemic treatments with pentavalent antimonials, pentamidine, interferon, allopurinol, rifampicin, dapsone, antimycotic azoles, and immunotherapy [6–10].

Despite the various therapies, the intralesional or systemic administration of pentavalent antimonials, such as sodium stibogluconate and meglumine antimoniate, is considered the standard therapy for cutaneous leishmaniasis [11,12].

Although these treatments have acceptable efficacy, they present problems such as their high cost, low adherence due to the prolonged use of intravenous or intramuscular injections, systemic toxicity, and, in some cases, resistance [13–19]. *Furthermore*, *pentavalent antimonial drugs are cardiotoxic (*prolongation of the Q-T interval *16%* ventricular repolarization disturbance *(25%) and arritmia 3*,*3%)*, *hepatotoxic (43%) and nephrotoxic (BUN increase 3*.*7%) and can cause pancreatitis (59*,*9%)*, *leukopenia (7*,*7%)*, *thrombocytopenia (7*,*1%)*, *arthralgia and myalgia (48*,*6%)* [20–22]. They are also contraindicated in pregnancy, lactation, infancy, people with hypersensitivity to the drug, and in some chronic diseases [2,11,23–31]

These drawbacks highlight the need to seek other treatment options for cutaneous leishmaniasis. Particularly, thermotherapy can overcome many of the limitations mentioned above, such as those related to adherence, cost, contraindications and side effects. Several studies have also reported that this disease can be cured with the application of local heat [32–34].

In addition, some studies have reported the use of caustic materials or cauterization with hot metal objects to treat the disease in rural communities [35–37].

Despite these previous reports, it should be noted that individual studies report some differences in the efficacy of this treatment because effectiveness was 48% in some studies [38], while it reached 94% in others [39]. In addition, some studies have been carried out with small sample sizes [40], affecting the statistical power of their findings.

Thus, the present study was conducted to evaluate the efficacy of thermotherapy versus pentavalent antimonials for the treatment of cutaneous leishmaniasis using controlled clinical trials published in the scientific literature.

## Materials and Methods

### Type of Study

Systematic review with a meta-analysis.

### PICO Question: Population Intervention Comparison Outcome (Outcome).

#### Population

Patients with a confirmed diagnosis of cutaneous leishmaniasis without mucosal involvement.

#### Intervention

Thermotherapy, local heat application (RF) at 50°C for 30 seconds in a single application or with repeated applications up to 4 weeks, with ThermoMed or ThermoSurgery, in the center of the lesion, active edges and area surrounding the lesion. Previously, asepsis was applied, and local anesthesia (usually with Xylocaine) was administered; after the intervention, antibiotics were applied to the lesion.

#### Comparison

Treatment with intramuscular, intravenous or intralesional pentavalent antimonials (sodium stibogluconate, meglumine antimoniate). In the studies that used two control groups with pentavalent antimonials with different application forms, both were added for the meta-analysis.

#### Outcomes

The primary outcome was the number of cured patients in each arm of the study, defined as the re-epithelization of lesions 3 months after starting treatment; secondary outcomes included treatment safety data (including pain, burning, itching, erythema, edema, inflammation, myalgia, generalized symptoms, arthralgia, and abnormal laboratory tests) reported in the individual studies.

### PRISMA Phases: Preferred Reporting Items for Systematic reviews and Meta-Analyses [41]

#### Identification or search for studies

An exhaustive search was conducted for controlled clinical trials published in the PubMed-Medline, Ovid-Medline, ScienceDirect, Embase, Wiley, SciELO, LILACS, and Web of Science databases; in addition, a search was conducted for clinical trials recorded in EBM Reviews—Cochrane Central Register of Controlled Trials, Cochrane Database of Systematic Reviews, EBM Reviews—Cochrane Methodology Register, EBM Reviews Full Text—Cochrane DSR, ACP Journal Club, and DARE as well as in other sources such as Springer Link, Jama Network and Oxford Journals.

The search terms used were "Cutaneous leishmaniasis" in combination with "Treatment", "Topical treatment", "Local treatment", "Local heat", "Heat therapy" and "Thermotherapy" and their counterparts in Spanish and Portuguese.

The thoroughness of the investigation was ensured by conducting a sensitivity search–i.e., without restricting it to MeSH (Medical Subject Headings) or DeCS (Health Sciences Descriptors) terms, allowing the identification of more studies than with the specificity search.

Examples of some syntax terms: i) (Cutaneous leishmaniasis[Title/Abstract]) AND Treatment[Title/Abstract]Filters:Clinical Trial, ii) cutaneous AND ("leishmaniasis"/exp OR leishmaniasis) AND ("topical"/exp OR topical) AND treatment/py, iii) cutaneous AND "leishmaniasis"/syn AND "topical"/syn AND treatment AND ([controlled clinical trial]/lim OR [randomized controlled trial]/lim)/py.

#### Screening or application of the inclusion criteria

Studies included were those with the search terms in the title, abstract or keywords, and if these were classified as experimental studies in the databases, unrestricted by the timing of the investigation or language of publication; the references of the articles were also reviewed to locate other manuscripts that were not found in the initial search, and the search option on "Related Articles" of PubMed was also used. Those articles that met these criteria were exported to the EndnoteWeb and Zotero programs to remove duplicates.

#### Selection or application of the criteria for exclusion and quality assessment of the studies

After reading the full texts that passed the screening phase, observational studies (which appeared to be classified as experimental by the databases) were excluded as well as controlled clinical trials evaluating interventions other than thermotherapy and articles with methodological quality problems such as non randomization.

### Data Extraction

The identification, screening, selection and inclusion stages were carried out by two researchers independently to ensure reproducibility, and it was determined *a priori* that differences were to be resolved by consensus or referral to a third party.

For the extraction of the study variables (year of publication, study location, type and number of patients per treatment, *Leishmania* species, and characteristics of the lesions), an Excel database was designed that was used independently by two researchers, resulting in an intra-observer and inter-observer kappa value of 1.00.

### Qualitative and Quantitative Synthesis

The characterization of the studies was based on the variables of time, place and person. The overall efficacy of thermotherapy and systemic treatment was estimated, and the healing rate of the two arms of the study was compared with the Z statistic.

A meta-analysis was performed for the relative risk using the random-effects model, given the heterogeneity found with the DerSimonian-Laird Q (chi-squared) test, and Galbraith and L'Abblé plots. Publication bias was studied with graphical methods using funnel plots and Egger plots and with analytical methods such as Begg (rank correlation) and Egger (weighted regression) statistical tests. Sensitivity analysis was performed using the random-effects model and influence plots to assess the contribution of each individual study to the overall outcome. The overall outcomes of the meta-analysis are presented using forest plots and a cumulative meta-analysis. The data were stored and analyzed in *Microsoft Excel* and in the Epidemiological Analysis of Tabulated Data Program of the Pan American Health Organization (EPIDAT) Version 3.0.

## Results

Table 1 shows the absolute number of studies found with the implementation of the Identification and Screening stages of the studies, noting the number of outcomes obtained with each of the search strategies in the eight databases. It should be explained that this strategy was applied in the Springer Link, Jama Network, and Oxford Journals databases, without the identification of additional studies. In Cochrane Central Register of Controlled Trials, using the search term "*Cutaneous leishmaniasis*", 226 studies were found, of which those evaluating thermotherapy appeared in the PubMed and OVID searches shown in Table 1.

**Table 1:** Absolute frequency of articles found during the stages of article identification and screening.

Source	Search terms & cutaneous leishmaniasis					
	Treatment	Topical treatment	Local treatment	Local heat	Heat therapy	Thermotherapy
**PubMed**						
No limits	3404	228	184	11	46	26
Title/Abstract	1197	62	15	5	7	10
Experimental (ECC)	175	6	2	2	2	4
**OVID**						
No limits	2390	7767	11601	4990	7646	12513
Title/Abstract/Keyword	19	9820	10579	5708	5469	7196
Experimental (ECC)	5	14	12	13	13	12
**ScienceDirect**						
No limits	6647	1309	2863	1065	1216	57
Title/Abstract/Keyword	343	34	19	4	4	4
Experimental (ECC)	47	12	3	3	3	4
**Embase**						
No limits	2249	423	167	15	61	70
Art & Clinical trial	154	53	13	3	5	8
Experimental (ECC)	48	34	10	3	4	8
**Wiley**						
No limits	3749	1220	1941	781	830	32
Title	73	7	1	1	3	0
Experimental (ECC)	47	5	1	1	3	0
**SciELO**						
No limits	125	32	19	1	0	1
Title	32	2	0	0	0	1
Experimental (ECC)	12	2	0	0	0	1
**LILACS**						
No limits	381	230	181	20	54	32
Title	49	73	11	4	8	1
Experimental (ECC)	5	19	3	1	2	1
**Web of Science**						
No limits	1590	200	96	14	30	21
Title	339	43	7	3	8	11
Experimental (ECC)	95	22	3	3	5	6

**Fig 1:**
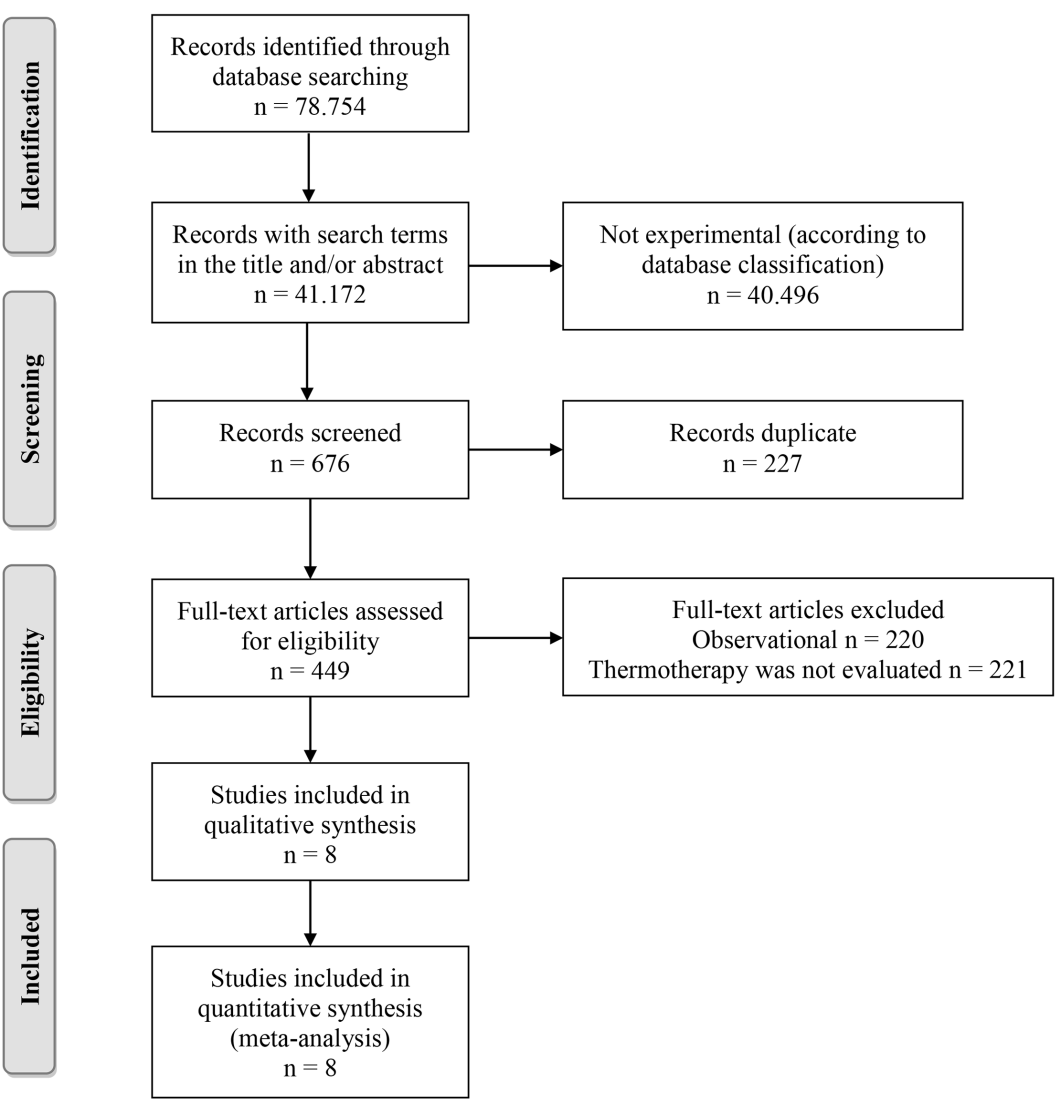
Article selection algorithm.

In total, 78,754 publications were identified, of which 41,172 included search terms in the title, abstract or both, and only 676 were classified as experimental studies or clinical trials in the databases; after removing duplicates, a complete reading of 449 studies was performed, of which only eight corresponded to controlled clinical trials evaluating the efficacy of thermotherapy (Fig 1). It should be noted that the PECET group published a study in which thermotherapy was compared with miltefosine [42], but this study was not included because the patients treated with thermotherapy were the same as those discussed in one of the included studies [20].

Four of the eight studies included were conducted in the Americas, three consisted of a military population, and four consisted of individuals over 17 years of age; overall, a combined population of 1289 patients was included, 622 undergoing thermotherapy and 667 undergoing systemic treatment; of these, different species of the parasite were evaluated according to the study location (Table 2).

**Table 2:** Description of the controlled clinical trials included in the study.

Author	Year	Country	Location	Age in years	# of Subjects	*Leishmania* species
Navin T [33]	1990	Guatemala	Soldiers	18–60	T^a^ = 22 G^b^ = 22	*L*. *(V) braziliensis L*. *(L) mexicana*
Reithinger R [43]	2005	Afghanistan	HealthNet International Khair Khana clinic		T^a^ = 108 G^b^ = 151	*L*. *(L) tropica*
Lobo I [40]	2006	Brazil	Bay Clinic	≥18	T^a^ = 16 G^b^ = 20	*L*. *(V) braziliensis*
Sadeghian G [44]	2007	Iran	Research Center for Skin Diseases and Leishmaniasis	≥5	T^a^ = 57 G^b^ = 60	No data
Aronson N [38]	2010	United States	Soldiers	≥18	T^a^ = 25 G^b^ = 2	*L*. *(L) major*
Safi N [45]	2012	Afghanistan	National Malaria and Leishmaniasis Control Program	≥4	T^a^ = 195 G^b^ = 195	*L*. *(L) tropica*
López L [20]	2012	Colombia	Soldiers	≥18	T^a^ = 149 G^b^ = 143	*L*. *(V) panamensis*, *L*. *(V) braziliensis*
Bumb R [39]	2013	India	Dermatology Center	≥ 5	T^a^ = 50 G^b^ = 50	*L*. *(L) tropica*

^a^T = Thermotherapy.

^b^G = Glucantime.

Regarding the qualitative synthesis of the meta-analysis, it was found that the main inclusion criteria were having a confirmed diagnosis of cutaneous leishmaniasis without mucosal involvement, having no previous treatment for the disease or no treatment within 8 weeks of the study, and having the laboratory tests within reference limits. In relation to the size and number of the lesions, there was variability among studies—the size was between 5 cm and 11 cm, and the number of lesions varied because some studies included only patients with less than four lesions, and four others included patients with fewer than 10 lesions. Exclusion criteria were being pregnant or lactating, having lesions in areas difficult for thermotherapy treatment, such as the eyes, fingers, near mucous membranes, face, or anal and urogenital orifices, and having hypersensitivity to pentavalent antimonials or local anesthesia, among others.

Additionally, only four studies did not reveal the randomization sequence [20,38,44,45], three presented the sample size calculation [38,43,45], one used a placebo as a control (three arms) [33], one study was focused on the immune response to the two treatments (systemic and thermotherapy) and evaluated clinical efficacy as healing at 28 days, as evidenced by a 50% decrease in the lesion size [40].

**Fig 2:**
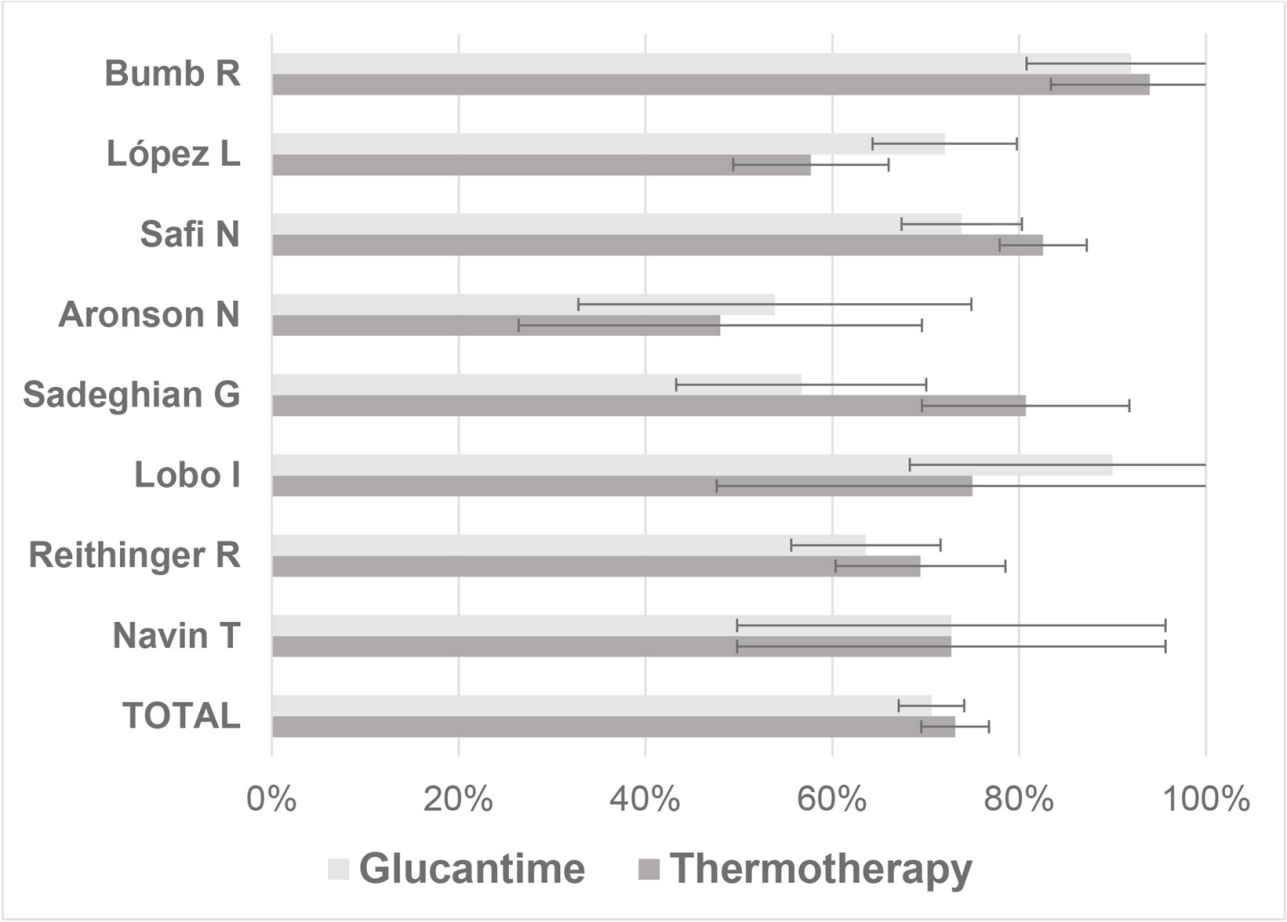
Efficacy of glucantime and thermotherapy for the treatment of cutaneous leishmaniasis (proportion with its 95% confidence interval).

In all of the studies, the efficacy obtained by thermotherapy was statistically equal to that of systemic treatment, with the lowest efficacy being reported in the study by Aronson, with 48% for thermotherapy and 53.8% for systemic therapy [38], and the highest efficacy was reported by the Bumb study for thermotherapy, with 94% and 92% for thermotherapy and systemic therapy, respectively [39] (Fig 2).

The overall efficacy of thermotherapy was 73.2% (95% confidence interval (CI) = 69.6–76.7%), whereas that of systemic treatment was 70.6% (95% CI = 67.1–74.1%) (Fig 2); between these rates of therapeutic efficacy, there were no statistically significant differences (CI for rate difference = -0.025; 0.076; Z statistic = 0.950; p-value = 0.342). Only two studies reported the efficacy of treatment per lesion, obtaining the best results with thermotherapy at 80.7% and 73% compared with systemic therapy at 55.3% and 59% [38,44].

**Fig 3:**
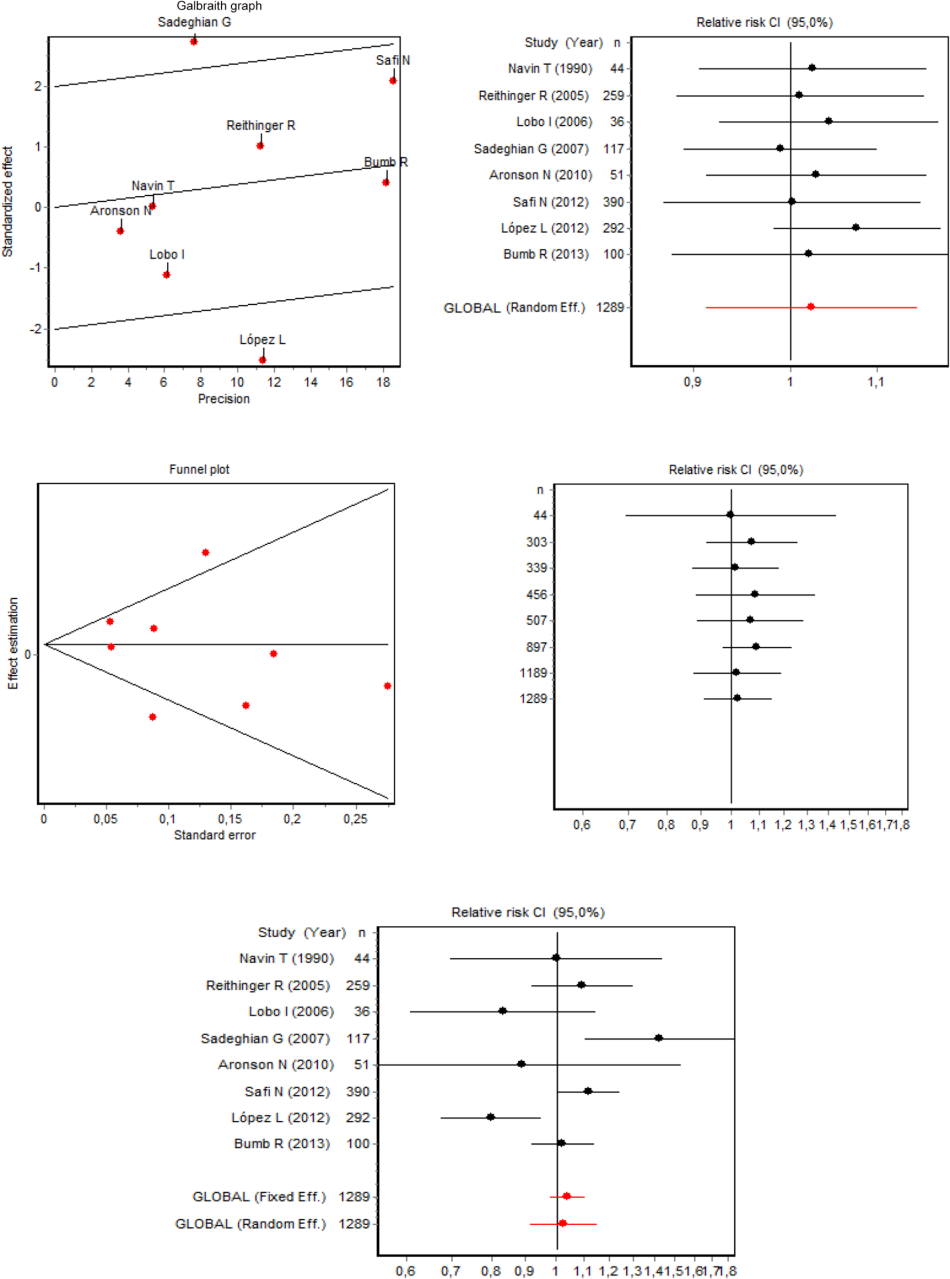
Meta-analysis of the efficacy of thermotherapy versus glucantime for the treatment of cutaneous leishmaniasis.

In the meta-analysis of efficacy, heterogeneity was found among studies (Galbraith plot of Fig 3) with the test for heterogeneity of DerSimonian and Laird's Q (chi-square) statistic = 19.168 (p-value = 0.0077) and RI coefficient = 0.6646 (proportion of total variance due to the variance between studies). There was no publication bias (funnel plot) (Fig 3), with the Begg test = 0.3712 (p-value = 0.7105) and Egger test = -0.4325 (p-value = 0.6805).

There were no sensitivity issues (influence plot) (Fig 3) because the removal of each study in successive stages did not change the conclusion found in the overall outcome of the meta-analysis. In this analysis, the change rates, between -3.37% and 5.04%, were found in the overall outcome due to the removal of each study, without changing the conclusion of the meta-analysis, as observed in the 95% confidence intervals for the relative risk (all intervals share values) (Table 3).

**Table 3:** Sensitivity analysis and meta-analysis with individual and combined results using the random-effects model.

Sensitivity analysis						
Omitted study	Year	N	Relative Risk (RR)	Confidence interval		Relative change (%)
				Lower limit	Upper limit	
Navin T [33]	1990	1245	1.056	0.91	1.17	0.15
Reithinger R [43]	2005	1030	1.011	0.88	1.16	-1.27
Lobo I [40]	2006	1253	1.044	0.93	1.18	1.91
Sadeghian G [44]	2007	1172	0.990	0.89	1.10	-3.37
Aronson N [38]	2010	1238	1.030	0.91	1.16	0.54
Safi N [45]	2012	899	1.003	0.87	1.15	-2.12
López L [20]	2012	997	1.076	0.98	1.18	5.04
Bumb R [39]	2013	1189	1.022	0.88	1.19	-0.23
GLOBAL		1289	1.024	0.91	1.15	
**Meta-analysis with individual and combined results**						
Study	Year	**N**	**RR**	**CI (95.0%)**	**% Fixed-effects Weight**	**% Random-effects Weight**
Navin T [33]	1990	44	1.00	0.70;1.44	2.73	7.08
Reithinger R [43]	2005	259	1.09	0.92;1.30	11.8	15.3
Lobo I [40]	2006	36	0.83	0.61;1.15	3.53	8.41
Sadeghian G [44]	2007	117	1.42	1.10;1.84	5.50	10.91
Aronson N [38]	2010	51	0.89	0.52;1.53	1.22	3.80
Safi N [45]	2012	390	1.12	1.01;1.24	32.12	19.59
López L [20]	2012	292	0.80	0.67;0.95	12.20	15.45
Bumb R [39]	2013	100	1.02	0.92;1.14	30.87	19.46
**Fixed effects**		**1289**	**1.03**	**0.97;1.10**		
**Random effects**		**1289**	**1.02**	**0.91;1.15**		

Random-effects meta-analysis shows that the efficacy of thermotherapy is statistically equivalent to the systemic treatment, with a relative risk of 1.02 (95%CI = 0.91; 1.15) (Table 3). The forest plot shows the overall meta-analysis relative risk (95% confidence interval) and that of the individual studies; it should be emphasized that the conclusions of the studies by Sadeghian [44] show evidence for the greater efficacy of thermotherapy, which is in contrast to the study by López [20] reporting a lower outcome efficacy; however, none of these studies affected the overall outcome or is significantly different from the combined outcome, as evidenced in the sensitivity analysis, which showed that it is not necessary to perform subgroup analysis (Fig 3). The cumulative meta-analysis shows how the addition of each study in successive stages does not change the conclusion of the study and improves the accuracy of the relative risk (Fig 3).

Regarding safety, the following effects were reported in patients with systemic treatment: transaminase elevation, erythema, edema, pruritus, fever, abdominal pain, pancreatitis, arthralgia, myalgia, headache, fatigue and cytopenia. However, in patients treated with thermotherapy, cellulitis, erythema, and pain at the lesion were found. It should be explained that the proportions of each effect described are not shown because these effects were not reported in most of the studies included.

## Discussion

In the present study, it was found that the efficacy of thermotherapy exceeded that of systemic treatment by 2.6%, although they were statistically similar, in a population of 1289 patients. These findings add to previous studies that, using an ethnomedical approach, have documented the way in which, in rural and indigenous communities in Latin America, empirical treatments are used with heat or caustic agents that are useful against cutaneous leishmaniasis [35–37]. In view of this, it is clear that thermotherapy can be added as a treatment option to existing schemes for the treatment of cutaneous leishmaniasis, particularly considering that it is a safer therapy, as demonstrated in the findings of the meta-analysis, being more cost effective and shorter and showing greater adherence by patients [45].

In addition to the above, it should be noted that thermotherapy reduces the adherence problems of other treatments, has no adverse systemic effects, requires no paraclinical tests, and can be used in patients with kidney, liver or heart diseases, pregnant women, and children, in other groups in which pentavalent antimonials or miltefosine cannot be administered and in particular cases in patients with human immunodeficiency virus infection/acquired immune deficiency syndrome [42,46]. Meanwhile, the safety problems of thermotherapy are easy to manage clinically because they only requires analgesia for a short period of time [42], in contrast to the adverse effects of systemic treatments whose management involves using greater and more complex health resources.

Nevertheless, in some scenarios, it has been argued that systemic treatment is better, basically by controlling the possibility that the cutaneous form would progress to mucocutaneous leishmaniasis, which depends on, among other factors, the infecting genus. In this way, skin infections by the subgenus *Viannia* have a high likelihood of progressing to mucosal leishmaniasis [47]. The discussion surrounding the above argument should be extended for the following reasons: i) the argument should only be widely applied to regions where there is a high prevalence of mucosal leishmaniasis rather than in countries where cutaneous leishmaniasis is the main presentation, such as in Colombia where the first presentation of the latter is less than 0.5% of cases; ii) in various scenarios, treatment coverage for cutaneous leishmaniasis is low (some have mentioned only 10%), mainly in rural areas, where nonmedical treatments predominate, although the prevalence of mucosal leishmaniasis is also low; iii) systemic treatment does not imply the absence of cases of mucosal leishmaniasis, only that systemic treatment is not completely necessary based on preventing cases of mucosal leishmaniasis [2,32–37,42]. Furthermore, systemic treatments have been shown to have reduced efficacy, attributable to the incomplete administration of the therapeutic regimens and adherence problems [20,33].

Other authors have questioned the duration of the therapeutic response in patients treated with thermotherapy; however, this issue can also be extrapolated to systemic treatments, which still lack high-quality evidence that can clarify the degree of parasite reactivation or its persistence, despite the clinical improvement observed. In this line of reasoning, some authors have indicated that systemic treatments induce long-term efficacy and are associated with the development of acquired immunity, although this has not yet been determined in thermotherapy. Aronson reported that a long-term cure was induced in soldiers treated with thermotherapy by showing that they remained without disease or evidence of infection one year after treatment, although this was a population that was outside of the endemic area at the start of treatment [38]; this limitation is overcome in the Bumb study, which reported that thermotherapy had long-lasting efficacy in patients who remained in endemic areas, up to 18 months after treatment, because parasites were not identified by PCR [39].

Among the genera and species causing the disease, it should be noted that the spontaneous cure of the infection or its progression to more severe forms varies; thus, in *Leishmania (L) major*, a spontaneous cure in 3 months occurs in 60–70% of cases; in *L*. *(L) tropica*, only 1% of cases results in a spontaneous cure; and *L*. (*V*) *braziliensis* is associated with greater severity, a chronic course, progression to a mucocutaneous form, and less spontaneous healing [48,49]. These data highlight the importance of adding these variables to the analysis of therapeutic efficacy, in conjunction with other clinical variables, such as lesion type, because this factor can alter the absorption of some topical medications. Thus, some topical agents are absorbed better in the typical ulcerative form of *L*. *(L) major* than in the nodular lesions that are more common in *L*. *aethiopica* and *L*. *donovani* [48].

The main limitations of this meta-analysis are related to the low number of studies included and the low number of patients analyzed in those studies, factors that prevented subgroup or meta-regression analyses, according to sociodemographic characteristics, parasite species, and the type, size and number of lesions. Additionally, some studies do not report adverse effects quantitatively, limiting the safety analysis. That studies were performed at diverse locations and during different time periods does not allow the standardization of research protocols for inclusion and exclusion criteria, the way in which the control was applied (two studies used an intralesional control [39,44], one used an intramuscular control [20], and another used an intravenous control), and other issues of methodological quality, such as sample size calculation and type of efficacy analysis. Moreover, meta-analysis statistics, particularly those that assess publication bias, have low power when used on a small number of studies [50,51].

Despite not controlling for these possible sources of heterogeneity among individual studies, the sensitivity analysis did not show that any of the studies would affect the overall outcome of the comparison of the thermotherapy efficacy against pentavalent antimonials.

Although the results obtained with this meta-analysis recommend the use of the thermotherapy in most cases of cutaneous leishmaniasis, it is very important carry out clinical trials in each region, in orden to improve the local scientific evidence available, in addition, to consider its peculiarities such as circulating parasite species, clinical features and forms of access to health services [2,52].

On the other hand, the massive use of thermotherapy is conditioned by the need for a specific device (Thermomed), by training who applied the treatment and care that requires the ulcer after burn like healings and local antibiotic use [2, 20].

## Conclusion

Thermotherapy presents statistically similar efficacy to that of systemic treatment, being safer, requiring a smaller number of treatments and no laboratory monitoring, improving adherence, and having a lower cost, among other things. Thus, thermotherapy can be recommended for use as a first-line therapy for the treatment of cutaneous leishmaniasis in areas where the prevalence of the mucocutaneous form is low and in patients where systemic therapies are contraindicated, such as those with kidney, liver and heart diseases, pregnants, and infants and patients with human immunodeficiency virus infection/acquired immune deficiency syndrome, among others. To this recommendation, efforts should be focused on patient education so that the signs and symptoms of complications due to progression to more severe forms can be identified.

## Supporting Information

S1 PRISMA Checklist This is the PRISMA 2009 Checklist Thermotherapy to treat cutaneous leishmaniasis. (PDF)
